# Epigenetic Reprogramming of CD4^+^ Helper T Cells as a Strategy to Improve Anticancer Immunotherapy

**DOI:** 10.3389/fimmu.2021.669992

**Published:** 2021-06-28

**Authors:** Elodie Renaude, Marie Kroemer, Christophe Borg, Paul Peixoto, Eric Hervouet, Romain Loyon, Olivier Adotévi

**Affiliations:** ^1^ University of Bourgogne Franche-Comté, INSERM, EFS BFC, UMR1098, Interactions Hôte-Greffon-Tumeur/Ingénierie Cellulaire et Génique, Besançon, France; ^2^ Centre Hospitalier Universitaire de Besançon, Centre d’Investigation Clinique, INSERM CIC 1431, Besançon, France; ^3^ Department of Pharmacy, University Hospital of Besançon, Besançon, France; ^4^ Department of Medical Oncology, University Hospital of Besançon, Besançon, France; ^5^ EPIGENEXP Platform, University of Bourgogne Franche-Comté, Besançon, France; ^6^ DImaCell Platform, University of Bourgogne Franche-Comté, Besançon, France

**Keywords:** epigenetics, CD4^+^ helper T cells, plasticity, tumor microenvironment, immunotherapy

## Abstract

Evidences highlight the role of various CD4^+^ helper T cells (CD4^+^ Th) subpopulations in orchestrating the immune responses against cancers. Epigenetics takes an important part in the regulation of CD4^+^ Th polarization and plasticity. In this review, we described the epigenetic factors that govern CD4^+^ T cells differentiation and recruitment in the tumor microenvironment and their subsequent involvement in the antitumor immunity. Finally, we discussed how to manipulate tumor reactive CD4^+^ Th responses by epigenetic drugs to improve anticancer immunotherapy.

## Introduction

### Distinct Roles of CD4^+^ Helper T Cells in Cancer

Tumor reactive CD4^+^ T cells are a heterogeneous subset that have different effector functions depending on the type of cytokines they produce (1, 2).

T helper 1 (Th1): Among CD4^+^ subsets, Th1 cells produce IFN-γ, IL-2, tumor necrosis factor α (TNFα) and express the T-box transcription factor (T-bet). Th1 cells play a well-defined role in antitumor protection by orchestrating cell-mediated immunity against cancer cells. Notably, these cells show the capacity to enhance tumor specific CD8^+^ T-cell generation, function, memory and survival (3–5). Additionally, the secretion of IFN-γ by Th1 cells can also promote CXCL9 and CXCL10 expression in the tumor microenvironment (TME) and therefore ensure the recruitment of CXCR3 expressing CD8^+^ T cells at tumor site (6, 7). Moreover, the production of IFN-γ by Th1 cells may enhance MHC class I and II expression at the surface of tumor cells, thus increasing tumor-derived peptide presentation. IFN- γ can also influence the polarization of macrophages toward proinflammatory M1 phenotype (8, 9). Emerging functions of Th1 cells also indicate their involvement in tumor angiogenesis inhibition, promoting cancer cell senescence, highly sensitive neoepitopes recognition, and protecting effector cytotoxic T lymphocytes (CTL) from exhaustion (4, 10). As a result, the presence of IFN-γ producing Th1 cells within the tumor microenvironment is associated with a good prognosis in several human cancers (11).

T helper 2 (Th2): Th2 cells produce IL-4, IL-5 and IL-13 and their differentiation is governed by the transcription factor GATA3 (12). Ambivalent roles have been described for these cells in the context of cancer. Indeed, their presence in the TME can be either beneficial or detrimental for patient survival (1, 2). In a mouse model wherein ovalbumin (OVA) is used as a specific tumor antigen, it has been shown that OVA-specific Th2 cells elicited a long-lasting antitumor response in mice. Indeed, IL-4 produced by memory anti-OVA Th2 cells directly stimulated natural killer (NK) cells cytotoxic activity against tumor (13–15). Moreover, the blockage of TGF-β signaling in CD4^+^ T cells of mice promoted a Th2 cell-differentiation program and reduced tumor growth. Indeed, IL4 produced by TGF-β receptor 2 (TGFBR2)-deficient CD4^+^ T cells in a mouse model of breast cancer could reprogram the tumor vasculature and triggered cancer cell hypoxia and death. These results support the antitumor role of Th2 cells (16). However, in human cancers, Th2 cells infiltration in the TME is commonly associated with poor clinical outcome (17, 18).

T helper 17 (Th17): Th17 cells are characterized by the master transcription factor retinoic acid receptor–related orphan receptor γt (RORγt). Th17 cells produce IL-17, IL-21, and IL-22. Their presence in the TME is either associated with a good or a poor prognosis (17, 19). In colorectal cancer, hepatocellular carcinoma, gastric and pancreatic cancer, Th17 cells polarization in TME was associated with an unfavorable prognosis whereas its presence in prostate, epithelial ovarian cancer or uterine cervical cancer was associated with a better clinical outcome (20–24)**. **The pro-tumoral roles of Th17 cells may be attributed to IL-17 proangiogenic functions. Indeed, IL-17 elicited VEGF production in colorectal cancer (25). Moreover, IL-17 signaling in tumor cells and tumor-associated stromal cells can lead to IL-6 production and induce STAT3 activation. STAT3 acts as an oncogenic factor by up-regulating pro-survival genes expression as well as metalloproteinases expression, thus favoring tumor invasion and metastases formation (26). IL-17 produced by Th17 cells may also influence the TME by promoting the recruitment of myeloid-derived suppressor cells (MDSCs) (27, 28).

Regulatory T cells (Treg): Treg cells express among others, the master transcription factor forkhead box P3 (FOXP3) and the high-affinity heterotrimeric IL-2 receptor (CD25). The presence of Treg cells in the tumor microenvironment is generally associated with poor clinical outcome in human cancers (11, 17, 19). These observations can be explained by their production of immunosuppressive cytokines (IL-10, TGF-β, IL-35) in the environment that can inhibit antitumor immunity (29–31). Treg cells have other mechanisms of humoral immunosuppression. For instance, Treg cells are highly dependent of IL-2 and can reduce its availability for effector T cells *via* their constitutive expression of CD25. Moreover, ATP can be converted into adenosine by CD39 and CD73 expressed on Treg cells. Adenosine is an immunomodulatory metabolite that can provide immunosuppressive signals to effector T cells and antigen-presenting cells (APC) *via* engagement of adenosine A_2A_ receptor (A_2A_R). The secretion of granzyme and perforin by Treg cells can also damage effector T cells. In addition, Treg cells display immunosuppressive functions by a contact-dependent mechanism through the expression of immunosuppressive receptors such as LAG-3 (lymphocyte-activation gene 3) or CTLA-4 (cytotoxic T-lymphocyte-associated protein 4). CTLA-4 binds to CD80 and CD86 on APC thus transmitting suppressive signals to these cells. Moreover, the co-stimulatory signals induced upon the binding of CD80 or CD86 to CD28 is prevented by CTLA-4, thus restraining the activation of effector B and T lymphocytes. Additionally, the interaction of LAG-3 with MHC class II on APC can suppresses dendritic cell maturation and immunostimulatory capacity (32–35). However, other studies in colorectal cancer associated Treg cells infiltration with a better prognosis (36, 37).

T follicular helper (Tfh): Tfh cells express the transcription factor B cell lymphoma 6 (BCL-6) and secrete CXCL13 and IL-21. Tfh help is essential for B lymphocytes activation, antibody production and memory formation. Tfh cells support the development of adaptive antitumor humoral responses and the formation of tertiary lymphoid structures (TLS) (38). Thus, Tfh cells infiltration in the TME is associated with a good prognosis in breast cancer and colorectal cancer (39, 40).

## Epigenetic Modifications in T Cells

**Figure 1:**
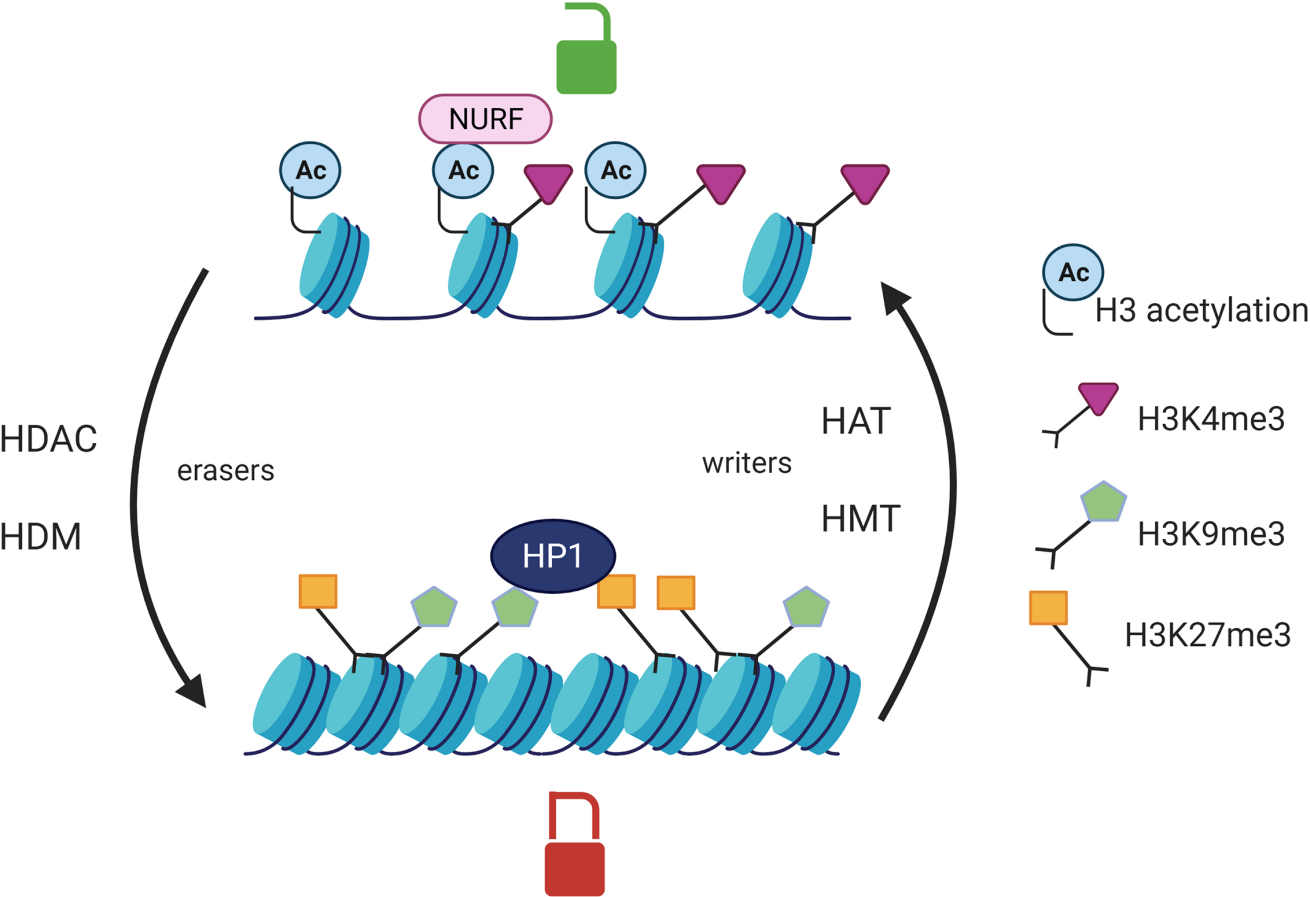
Post translational histone modifications and their effects on transcription. Histone acetylation catalyzed by HAT is associated with an open chromatin whereas HDAC erase this mark and are responsible for chromatin compaction. HMT add methyl groups on the lysines of histone H3 protein whereas HDM catalyze the opposite reaction (eraser). The effect of histone methylation on transcription is variable. HP1 (heterochromatin protein 1) can recognize H3K9me3 or H3K27me3 and is responsible for chromatin silencing. NURF (nucleosome remodeling factor) recognizes the histone mark H3K4me3 and is associated with a chromatin permissive to transcription. HAT, histone acetyltransferases; HDAC, Histone deacetylases; HMT, Histone methyltransferases; HDM, histone demethylases.

Epigenetic modifications such as DNA methylation and post-translational histone modifications can regulate gene expression by modulating chromatin accessibility to transcription factors. Epigenetics is a reversible process as there are enzymes catalyzing the apposition of the post translational modifications such as histone methyltransferases (HMT) and histone acetylases (HAT) (epigenetic writers) and enzyme responsible for the demethylation and deacetylation of histones (HDAC) which are referred to as epigenetic eraser (**Figure 1**). Histone acetylation is associated with a permissive chromatin state whereas histone deacetylation generates a close chromatin state which is transcriptionally inactive. Histone methylation can be either favorable or unfavorable to transcription depending on the number and the position of the methyl groups on the histone tail. Trimethylation of the lysine 9 of histone 3 (H3K9me3) or H3K27me3 can be recognized by the heterochromatin protein HP1 which is responsible for chromatin silencing. On the contrary, H3K4me3 is positively correlated with gene transcription as it is recognized by NURF (nucleosome remodeling factor). The NURF complex is associated with a permissive chromatin and is enriched at promoter transcription starting sites (41, 42). The combination of post-translational histone modifications is called the histone code and overall defines chromatin accessibility to transcription factors (43) (**Figure 1**). DNA methylation on cytosines occurs preferentially at CpG islands which are composed of a high GC base density and located at promoter or distal cis-regulatory elements, like enhancers. DNA methylation is catalyzed by DNA methyltransferases (DNMT) and is associated with gene silencing (44). At the post transcriptional level, noncoding RNA (ncRNA) can target complementary mRNA epigenetic and impair mRNA translation. Indeed, the double strand complex is then recognized by the RNA-induced silencing complex (RISC) and can result in the cleavage of the targeted mRNA or the inhibition of its translation (45).

### Epigenetics in CD4^+^ T Cells Differentiation and Plasticity

#### Epigenetics Regulates CD4^+^ T Cells Differentiation

**Figure 2:**
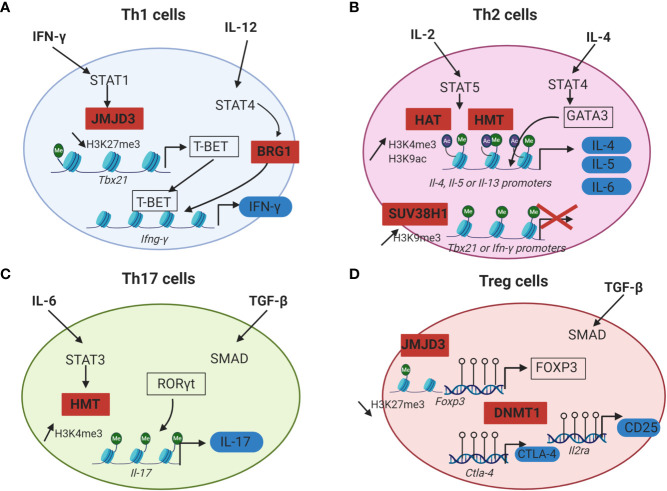
Epigenetic mechanisms that regulate CD4^+^ T cells differentiation. For each CD4^+^ T cell subset, cytokines initiating Th cells differentiation are in bold, lineage specific transcription factors are framed in black and effector proteins are surrounded in blue. Epigenetic enzymes regulating CD4^+^ T cells differentiation are in red squares. **(A)** IFN-γ and IL-12 drive Th1 cells differentiation by inducing STAT1 and STAT4 activation. STAT1 recruits JMJD3 (H3K27me3 histone demethylases) at the promoter of *Tbx21.* This promotes the loss of the repressive mark H3K27me3 and allows T-BET expression. STAT4 induces the recruitment of a chromatin remodeling complex (BRG1) at the locus of *Ifn-γ* which is responsible for a chromatin permissive to transcription. T-BET can then bind to the *Ifn-γ* promoter and induce its transcription. **(B)** In Th2 cells, IL-2 signals through STAT5 and triggers the recruitment of HAT and HMT responsible for the increase of the permissive marks H3K9ac and H3K4me3 at *Il-4, Il-5* and *Il-13* gene loci. IL-4 plays a role in inducing STAT6 activation, which upregulates the expression of the master regulator GATA3. GATA3 can then bind to the promoters of *Il-4, Il-5* and *Il-13* and induce the transcription of these effector cytokines. In Th2 cells, Th1 associated genes are silenced by the HMT SUV39H1 which increases the repressive mark H3K9me3 at the loci of *Tbx21* and *Ifn-γ* and allows Th2 lineage stability. **(C)** During Th17 differentiation, IL-6 induces STAT3 activation and binding to the *Il-17* promoter. STAT3 then recruits histone modification enzymes at the *Il-17* locus, resulting in an increase of the permissive marks H3K4me3 and chromatin accessibility. RORγt can thus bind to the *Il-17* promoter and induce IL-17 expression. **(D)** Treg cells differentiation from naïve CD4^+^ T cells requires TGF-β. The expression of the master transcription factor FOXP3 as well as the immune checkpoint CTLA-4 and the IL-2RA (CD25) is dependent of DNA demethylation at the loci of these genes by DNMT1. Empty circles on DNA: unmethylated CpG island.

The cytokines present in the microenvironment during TCR activation drive a cascade of signalization and activate transcription factors, which in turn modulate epigenetic enzymes leading to the CD4^+^ T cell polarization. Epigenetic modifications trigger the chromatin accessibility to lineage specific master transcription factors (T-bet, GATA3, RORγt, FOXP3) responsible of CD4^+^ T cells commitment (**Figure 2**). There is an increase of DNA demethylation and permissive histone modifications at lineage specific genes loci as well as a decrease of repressive histone marks which allow the expression of specific transcription factors and effector cytokines (46, 47). Indeed, the CD4^+^ T cell profile is dependent of the cytokines produced in the environment by innate immune cells following danger signals (48, 49).

Th1 cells differentiation is dependent of IFN-γ and IL-12. IFN-γ signaling in the activated CD4^+^ T cells triggers the activation of STAT1 signalization pathway and thus promotes T-bet expression. T-bet further increases the IFN-γ production and IL-12 receptor expression in the differentiating cells. IL-12 signaling induces STAT4 activation which promotes IFN-γ production by an epigenetic mechanism. Indeed, STAT4 bound to the *Ifn-γ* promoter in Th1 cells and recruits a chromatin remodeling complex called Brahma related gene 1 (BRG1), that remodels the nucleosomes and enhances the transcription of IFN-γ (**Figure 2A**) (50). Moreover, chromatin immunoprecipitation (ChIP) assays revealed that the level of permissive histone H4 acetylation marks at the *Ifn-γ* promoter of activated CD4^+^ T cells cultured under Th1 polarizing conditions (IL-12 and anti-IL-4) were higher compared to Th2 cells or undifferentiated Th cells. ChIP assays also indicated that differentiation into Th1 cells was accompanied by a decrease of HDAC-Sin3A recruitment at the *Ifn-γ* locus. By contrast, in undifferentiated CD4^+^ T cells, the level of HDAC-Sin3A was enhanced at this locus, thus preventing the formation of stable H4 acetylation marks. The transduction of T-bet into undifferentiated CD4^+^ T cells was sufficient to reduce the level of HDAC-Sin3A at the *Ifn-γ* locus and was associated with a gain of H4 acetylation across this locus as well as IFN-γ production. Taken together, these results indicated that the loss of HDAC-Sin3A at the *Ifn-γ* locus in effector Th1 cells was actively regulated by T-bet thus allowing the expression of IFN-γ (51). T-bet not only induces Th1 differentiation, but also prevents the differentiation toward the other subsets of CD4^+^ T cells by repressing their master transcription factors (GATA3 and RORγt) (52, 53).

The inhibition of Th1 associated genes in Th2 cells is mediated by epigenetics. In Th2 cells, the expression of IFN- γ is inhibited by the deposition of the repressive histone mark H3K27me3 to the *Ifn-γ* locus by EZH2 (54). Moreover, the silencing of Th1 gene loci was reported to be associated with an increase of the repressive histone modification H3K9me3 at these loci. The HMT SUV39H1 is responsible for H3K9 methylation. The deposition of H3K9me3 then recruits the heterochromatin protein 1α (HP1α) and promotes transcriptional silencing with the formation of heterochromatin at the promoter of Th1 specific genes, thus allowing Th2 lineage stability (55).

Regarding the initiation of Th2 differentiation, IL-2 and IL-4 are essential for Th2 cells generation from naive CD4^+^ T cells. IL-2 signals through STAT5 whereas IL-4 plays a role in inducing STAT6 activation, which upregulates the expression of the master regulator GATA3 (56). ChIP analysis of Th2-primed cells revealed that STAT5A could bind the *Il-4* gene but failed to do so in cells primed under Th1 conditions or with anti-IL-2. Upon activation and differentiation of human Th2 cells from naïve CD4^+^ T cells, the permissive marks H3K9 acetylation and H3K4me3 were increased at *Il-4, Il-5* and *Il-13* gene loci. This chromatin remodeling occurring at most of the Th2 cytokine gene loci revealed the important part played by epigenetics in Th2 cell differentiation (**Figure 2B**) (57).

Epigenetics also plays a key role in the differentiation of Th17 cells [for a review: Renaude et al. (58)]. TGF- β, IL-6, IL-21 and IL-23 are required for Th17 differentiation from naive T cells. Briefly, TGF-β activates the Smad signaling pathway whereas IL-6 induces STAT3 activation (59). STAT3 binding to the *Il-17* promoter correlates with an increase of the permissive marks H3K4me3 at the *Il-17* locus (60) (**Figure 2C**). The deposition of permissive or repressive histone marks at Th17 specific gene loci regulates the chromatin accessibly to these genes and is therefore essential for the expression of Th17 specific cytokines IL-17 and IL-21 (61).

The comparison of the DNA methylation landscape between conventional CD4^+^ T cells and Treg cells revealed that DNA of Treg cells is globally hypomethylated. This observation correlated with the expression of genes vital for Treg cell function, such as *Foxp3, Ctla4* and *Il2ra* (62, 63) (**Figure 2D**). Moreover, conditional deletion of Dnmt1 in CD4^+^ T cells of mice resulted in a decrease of the number and the immunosuppressive function of peripheral Treg cells, leading to lethal autoimmunity (64). Additionally, JMJD3, a histone H3K27 demethylase, was found to skew CD4^+^ T cell differentiation *in vivo*. The specific ablation of JMJD3 in the T cells of mice promoted Th2 and Th17 differentiation and inhibited Th1 and Treg cells polarization. Mechanistically, ChIP assays revealed that JMJD3 KO T cells were associated with a decrease of the permissive mark H3K4me3 at the *Foxp3* promoter as well as an increase of the repressive mark H3K27me3 at the *Ifn-γ* promoter (65).

Tfh cells differentiation is also regulated by an epigenetic mechanism. Indeed, in a model of acute viral infection in mice, an increased level of the histone mark H3K27me3 was observed in Tfh cells compared to Th1 cells. The ablation of EZH2 (H3K27 HMT) in virus-specific CD4+ T cells resulted in an impaired differentiation of Tfh cells. EZH2 was found to regulate the chromatin accessibility of Tfh lineage associated genes such as *Bcl-6* and *Il-21* (66, 67). These data support evidence for the implication of epigenetics in the formation of CD4^+^ helper T cells differentiation.

#### Epigenetic Regulation of CD4^+^ T Cells Plasticity

CD4+ T cells are characterized by plastic capacities and can transdifferentiate into other subsets and this phenomenon is mediated by epigenetics. First evidence of CD4^+^ T cell plasticity was brought by the existence of cells expressing simultaneously genes that were specific of other CD4^+^ T cell lineages. For example, the co-expression of cytokines specific of Th17 and Th2 cells, IL-17 and IL-4, was identified in human circulating CD4^+^ T cells and revealed the potential plasticity of Th17/Th2 cells (68). Moreover, Th17 cells were found to convert into Th1 cells with the production of IFN-γ. In some cases, IL-17 expression was maintained in these cells whereas Th1 arising from Th17 cells could also totally extinguished IL-17 expression, indicating a progressive process and a terminal differentiation toward the Th1 phenotype. The conversion of Th17 cells into Treg cells was also observed in mouse and human. These cells expressed the lineage transcription factor FOXP3 as well as IL-17 and showed immunosuppressive capacities with the expression of IL-10 (69, 70).

Among the CD4^+^ helper subsets, Th17 cells elicit the greatest plastic capacities and can eventually convert into the other subsets under specific conditions. This particularity can be attributed to the fact that the expression of the cytokines and transcription factors defining Th17 cell lineage is unstable. Indeed, ChIP sequencing assays performed on the different CD4^+^ T cell subsets have revealed the existence of a “poised” chromatin state in Th17 cells. This was characterized by the presence of both repressive and permissive histone marks at other lineage specific loci and was associated with an absence of DNA methylation. These results indicate that the expression of other lineage specific genes is not strongly repressed in Th17 cells (71, 72). Moreover, the expression of the Th1 cell-like phenotype induced by the *in vitro* culture of Th17 cells with IL-12 was correlated with a decrease of the permissive marks H3K4me and histone acetylation at the *Il-17* locus and an increase in these marks at the *Ifn-γ* locus These modifications resulted in an increased production of IFN-γ by these cells and a reduction of IL-17 secretion (73). The implication of epigenetics in CD4^+^ T cell plasticity was confirmed by Li et al., (65), as *Jmjd3* deficiency in mice restrains the plasticity of the conversion of Th2, Th17 or Treg cells to Th1 cells (65).

The activity of epigenetic enzymes can also be modulated by the metabolism and can therefore affect CD4^+^ T cell plasticity. As an example, the metabolite 2-hydroxyglutarate is an inhibitor of ten-eleven translocation (TET) enzymes, which are implicated in DNA demethylation. The reduction of 2-hydroxyglutarate level in Th17 cells after aminooxy-acetic acid treatment led to the diminution of *Foxp3* promoter methylation, therefore stabilizing FOXP3 expression and driving the Th17/Treg plasticity (74, 75). Epigenetic enzymes activity is dependent of the availability of metabolites like S-adenosylmethionine (SAM), a donor of the methyl group needed for DNA and histone methyltransferases activity and can therefore be affected by changes in their availability. An excess of SAM in the microenvironment may increase gene silencing by enhancing DNA methylation and affect CD4^+^ T cell plasticity (76).

### Epigenetics in CD4^+^ T Cells Memory

Epigenetics is also involved in the maturation process of CD4^+^ T cells and the generation of the immune memory. Durek et al., (77), studied the epigenetic landscape of naive, central memory, effector memory, and terminally differentiated CD4^+^ T cells from the blood (77). They performed extensive epigenetic profiling: DNA methylation analysis, chromatin accessibility studies and ChIP sequencing for H3K4me1, H3K4me3, H3K9me3, H3K27ac, H3K27me3, and H3K36me3 as well as sequencing of coding and non-coding RNA of these cells. Their results showed a global loss of DNA methylation upon transition from the naive to the memory stages. Progressive changes in the transcriptomes and in the DNA accessibility profiles suggested that the differentiation of CD4^+^ T memory subsets support a linear model. Moreover, the authors identified important factors that drive or maintain the CD4^+^ memory phenotype. Among these factors, they identified many non-coding RNAs (ncRNAs), which were differentially expressed in naive and memory T cells. The transcription factor FOXP1 might act as an important regulator for the naive to memory transition as its expression was higher in naive CD4^+^ T cells compared to the other subsets of memory T cells. Foxp1 expression was repressed by DNA methylation in memory T cells.

### Involvement of Epigenetic in the Control of Tumor Reactive CD4^+^ T Cells

It has been demonstrated that epigenetic modulation of CD4^+^ T cells polarization at tumor site might influence cancer patients’ outcome. Since CD4^+^ T cells polarization is epigenetically regulated, characterization of immune cells infiltration at tumor site may be assessed by epigenetic analysis. Among Treg cells, two subsets have been defined based on their developmental origin. Thymus-derived natural Tregs (nTregs) show a stable expression of FOXP3 associated with DNA demethylation, whereas peripherally induced Tregs (iTregs) cells do not express FOXP3 constitutively. In mouse tumor models and primary human tumors, analysis of DNA methylation patterns at the *Foxp3* locus as well as functional studies revealed that Treg cells infiltrating in the TME mainly corresponded to nTreg and not iTreg cells (78). The functionality of conventional helper T cells as well as Treg cells in the TME can be modulated by epigenetics. Genome wide DNA methylation landscape of tumor infiltrating and blood CD4^+^ T cells from glioblastoma patients revealed differentially methylation pattern. The methylation changes were associated with transcriptomic changes for 341 genes in CD4^+^ tumor infiltrating T cells compared to blood. This study revealed that the TME may induce epigenetic alterations in tumor infiltrating CD4^+^ T cells (79). Depletion of tumor-associated macrophages (TAMs) in pancreatic cancer could also reprogram the epigenetic profile of tumor infiltrating CD8^+^ and CD4^+^ T cells. In a model of pancreatic cancer in mice, TAMs were depleted using trabectedin and the cytokine and epigenetic profile of T cells were assessed. Tumor infiltrating CD4^+^ T cells displayed a regulatory phenotype with high IL-10 expression and low IFN-γ production. On the contrary, in trabectedin-treated mice, the permissive histone mark H3K4me3 was decreased and the repressive mark H3K27me3 was increased at the *Il-10* promoter resulting in low IL-10 expression. This study highlighted the role of TAMs in modulating the epigenetic profile of tumor infiltrating CD4^+^ T cells towards a pro-tumoral phenotype (80). In melanoma patients, EZH2 expression was increased in Treg cells infiltrating the tumor compared to Treg cells from the peripheral blood. Genetic deletion of *Ezh2* in Treg cells from tumor-bearing mice reduced FOXP3 expression and modulated their functionality. Indeed, *Ezh2*-deficient tumor infiltrating Treg cells showed an increased production of the pro-inflammatory cytokines TNF-α, IFN-γ, and IL-2 and a reduced expression of IL-10. This observation was correlated with an enhanced recruitment and function of CD8^+^ and CD4^+^ effector T cells in the TME and led to the tumor elimination in mice (81). Epigenetics is also involved in CD4^+^ T cells exhaustion in the TME. The inhibitory receptor programmed cell death-1 (PD-1) was found to be epigenetically regulated in tumor-reactive lymphocytes. Indeed, upon T cell activation, the chromatin organizer special AT-rich sequence-binding protein-1 (SATB-1) inhibited PD-1 expression by recruiting a nucleosome remodeling deacetylase (NuRD) complex to *Pdcd1* regulatory regions. Inhibition of STAB-1 in CD4^+^ and CD8^+^ T cells increased PD-1 expression and resulted in the loss of their effector activity more rapidly than wild-type lymphocytes. The transfer *type* -deficient tumor-reactive CD4^+^ T cells in Lewis Lung Carcinoma-bearing mice, resulted in a decreased survival rate. Therefore SATB-1 functions to prevent premature T cell exhaustion by regulating *Pdcd1* expression upon T cell activation (82). Moreover, in colorectal cancer patients, other immune checkpoint inhibitors have been found to be epigenetically regulated in CD4^+^ and CD8^+^ tumor infiltrating lymphocytes. The promoter regions of *T-cell immunoglobulin and mucin-domain containing-3 (Tim-3), Ctla-4, Pdcd1, Programmed cell death-ligand 1 (Pd-l1), Tox* and *Tox2* were highly demethylated whereas the promoter of *Lag-3* was highly methylated. These results correlated with the transcriptional upregulation of Tim-3, Pd-1, Pd-l1, Ctla-4, Tox and Tox2 in CD4^+^ and CD8^+^ infiltrating colorectal cancer (83).

CD4^+^ T cells plasticity in the TME can be modulated by an epigenetic mechanism. Indeed, tumor associated macrophages (TAM) residing at tumor site can release exosomes containing miRNA. Microarray analysis of these exosomes revealed the presence of miR-29a-3p and miR-21-5p. *In vitro*, the treatment of CD4^+^ T cells by these miRNAs suppressed STAT3 and induced Th17 toward Treg commitment. These miRNAs can therefore lead to the development of Treg in the TME and thus engender an immunosuppressive context that facilitates cancer progression and metastasis (84). The plasticity of Th17 clones generated from human tumor-infiltrating lymphocytes after *in vitro* stimulation with OKT3 and irradiated allogeneic peripheral blood mononuclear cells was also demonstrated. Following expansion, the level of IL-17 production by these cells dropped whereas the expression of TNF-α, IFN-γ, IL-10 and TGF-β increased. Real time PCR analysis revealed that T-bet and Foxp3 expression gradually increased in Th17 clones with the expansions. The level of DNA methylation at the *Foxp3* promoter in expanded Th17 cells decreased significantly with increasing stimulation and expansion cycles. These results indicated that the expansion of Th17 clones from tumor infiltrating lymphocytes promoted their conversion into mixed phenotypes that expressed FOXP3 and produced IFN-γ. These phenotypic changes resulted from the epigenetic reprogramming of lineage-specific genes (70). The expression of chemokines responsible for the attraction of CD4^+^ T cells at the tumor site can be modulated by epigenetics. Indeed, EZH2 (H3K27 HMT) as well as DNMT1 were found to repress the expression of the Th1-attracting chemokines CXCL9 and CXCL10 in primary ovarian cancer model. Th1 cells express the chemokine receptors CXCR3 and can thus be recruited by CXCL9 and CXCL10 enriched environments (56). The increase of the repressive mark H3K27me3 and DNA methylation at the promoter of *Cxcl9* and *Cxcl10* inhibited their expression and prevented Th1 cell infiltration in the TME. Inhibition of EZH2 and DNMT1 by chemical inhibitors restored the expression of CXCL9 and CXCL10 and increased effector T cell tumor infiltration (85).

Th1 cells recruitment in the TME can also be induced by the expression of endogenous retroviruses (ERV) by tumor cells. ERV originate from ancient retroviruses whose sequences are now permanently integrated in the genome. Their expression is normally repressed by DNA methylation. Increased ERV expression by DNA methyltransferases treatment can trigger the activation of viral defense pathways. Indeed, ERV can be recognized by Toll-like receptors (TLR) on the surface of innate immune cells and activate viral defense pathway. This induces IFN-γ production and triggers lymphocytes infiltration that may further favor antitumoral responses (86, 87).

## How Manipulation of CD4^+^ T Cells by Epigenetic Therapies Can Be Used as a Strategy to Improve Anticancer Immunotherapy in Solid Tumors?

**Figure 3:**
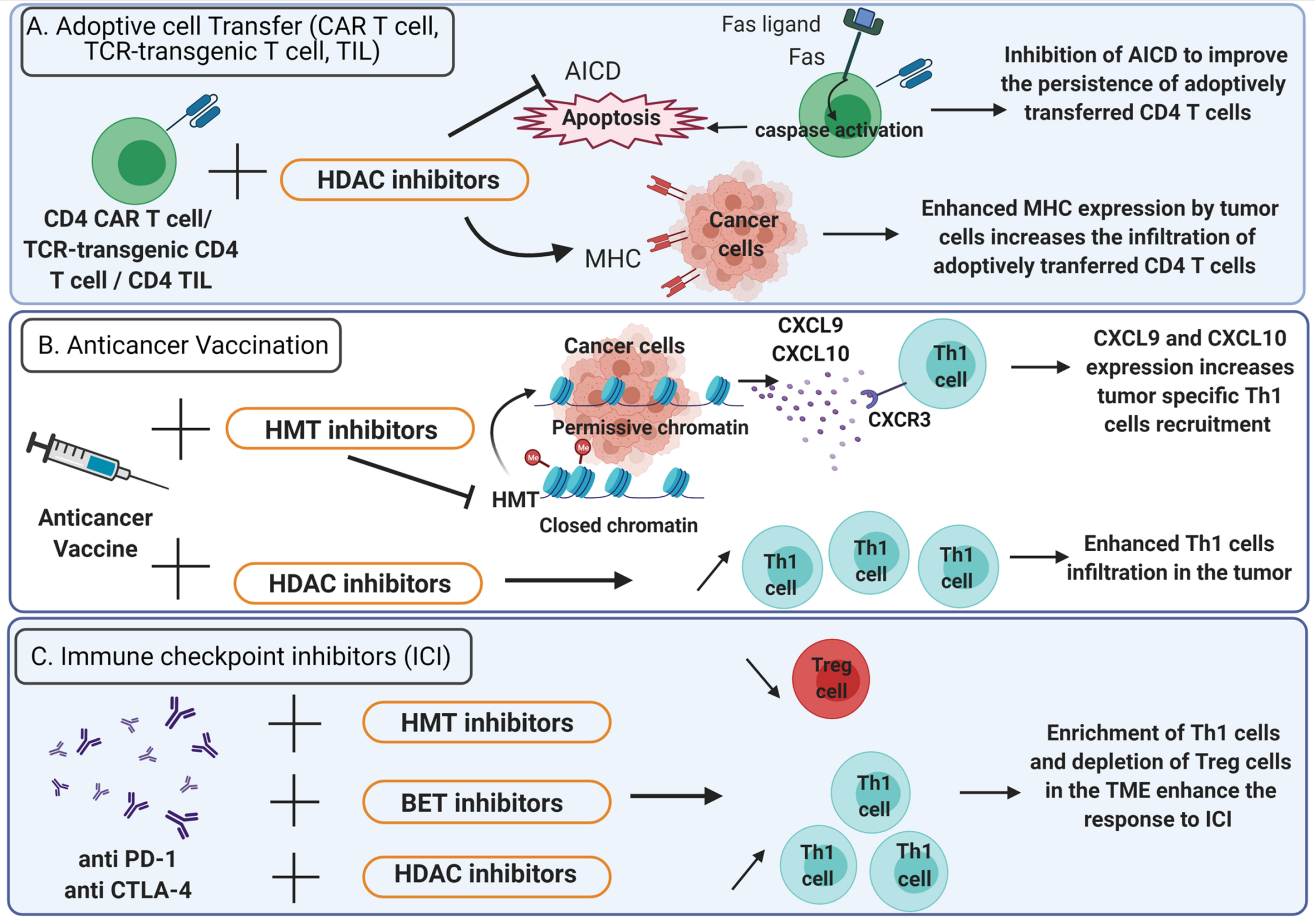
Epigenetic therapies potentiate the efficacy of anticancer immunotherapy. **(A)** Adoptive cell transfer. To improve the persistence of adoptively transferred CD4^+^ CAR T cells, TCR-transgenic CD4^+^ T cells or CD4^+^ TIL in the TME, activation induced cell death (AICD) can be inhibited using HDAC inhibitors (bar-headed line). AICD is mediated by Fas-Fas ligand interactions and triggers the activation of caspases that lead to apoptosis (solid arrows). HDAC inhibitors also enhance MHC expression on tumor cells thus increasing the infiltration of adoptively transferred CD4^+^ T cells. **(B)** Anticancer vaccination: HDAC inhibitors favor Th1 cells enrichment in the TME. HMT inhibitors promote the expression of CXCL9 and CXCL10 by tumor cells and enhance the recruitment of Th1 cells expressing CXCR3. **(C)** Immune checkpoint inhibitors: the efficacy of anti-PD-1 and anti-CTLA-4 is enhanced by HDAC, HMT and BET inhibitors which favor a TME enriched in Th1 cells and depleted in Treg cells. TME, Tumor microenvironment; TIL, Tumor infiltrating lymphocytes; CAR T cells, chimeric antigen receptor T cells; HDAC, Histone deacetylase; HMT, histone methyltransferase; BET, bromodomain and extra-terminal domain family protein.

Evidence support the rational to stimulate CD4^+^ T cell responses for anticancer immunotherapies (88–90). Considering the important role played by epigenetics in CD4^+^ T cells differentiation and plasticity, strategies of epigenetic reprogramming gained growing interest to promote appropriate antitumor CD4^+^ helper T cells in the TME (**Figure 3**).

Adoptive transfer of tumor specific CD4^+^ lymphocytes has proved its efficacy in the treatment of cancer (91–93). However, it is uncertain whether CD4^+^ T cell polarization status, acquired during *in vitro* stimulation, can sustain in the TME after T cell infusion. Thus, an epigenetic treatment could be administered to preserve CD4^+^ T cells polarization in the TME. It has been shown that epigenetic modulators can improve the efficacy of adoptive CD4^+^ T cell therapies by increasing MHC expression on tumor cells (**Figure 3A**). Several HDAC inhibitors (HDACi) such as chidamide, entinostat, vorinostat and CXD101 have been found to upregulate MHC class II expression in various cancer cell lines (94). Moreover, the expression of a constitutively active STAT5 variant (CASTAT5) by CD4^+^ chimeric antigen receptor (CAR) T cells targeting the B cell antigen CD19 could improve the polyfunctionality, expansion and persistence of these cells in the TME by an epigenetic mechanism. Indeed, CASTAT5-transduced CD4^+^ T cells displayed a genome-wide transcriptional and epigenetic remodeling. Assay for transposase-accessible chromatin using sequencing (ATAC-seq) performed on CASTAT5 CD4^+^ T cells identified an increased chromatin accessibility at the gene loci of *Il-4, Il-13, Il-9*, and *GzmB* but not at the *Ifn- γ* locus. These results were concordant with gene transcription and protein expression profiles and indicated that persistent STAT5 activation reprogrammed the epigenetic landscape of CD4^+^ T cells to drive polyfunctionality. In B cell lymphoma-bearing mice, the adoptive transfer of CD19 CAR T cells resulted in only transient tumor regression whereas the adoptive transfer of CASTAT5 CD19 CAR T cells was curative to nearly all mice (95). In adoptive cell transfer, the induction of CD4^+^ T cells persistence at tumor site represents a critical issue (96). For example, Fas-Fas ligand dependent activation-induced cell death (AICD) can be prevented by HDACi. Indeed, in a mouse model, the use of HDACi inhibits AICD of CD4^+^ T cells in the TME (97) (**Figure 3A**). Recently, it has been demonstrated that a low dose decitabine priming could enhance the persistence and the antitumor activity of CD4^+^ CAR T cells. Indeed, the treatment of CD4^+^ and CD8^+^ CAR T cells with the DNA methyltransferase inhibitor decitabine could upregulate the expression of memory and proliferation associated genes and downregulate the expression of T cell exhaustion related genes: *Lag-3* and *Ctla-4*. *In vitro*, after co-culture with Raji tumor cells, the proliferation of CD4^+^ CAR T cells treated with decitabine was enhanced as well as the expression of IL-2, IFN-γ, TNF-α, Perforin, Granzyme A and Granzyme B. Upon activation, CD4^+^ CAR T cells treated with decitabine showed a significative increase of the expression of the chemokines CXCL8, CXCL10, CXCL1, CCL3 and CCL1. *In vivo*, decitabine pre-treated CD4^+^ and CD8^+^ CAR T cells targeting the CD19 antigen resulted in complete tumor regression in mice and persisted longer than untreated CAR T cells (98).

The CD4^+^ T cell help is critical for the success of anticancer vaccines (99, 100). Th1 cells are essential for the induction of an effective antitumor response after vaccination (4). Thus, the administration of epigenetic therapies to modulate the CD4^+^ T cells polarization in the context of anticancer vaccine can be considered (**Figure 3B**). In preclinical models of triple-negative breast cancer 4T1 and colon carcinoma MC38, Hicks et al. (101), demonstrated that the addition of entinostat, a class I HDACi, to vaccine significantly improved the tumor control. The entinostat treatment reprogrammed the TME toward an inflamed phenotype with an enhanced expression of proinflammatory genes (IFN-γ, TNF-α) and a decrease of regulatory T cells in the TME (101) (**Figure 3B**). This study highlights the promising use of epigenetic therapeutics to improve the response to anticancer vaccines.

The immune checkpoint inhibitors (ICI) such as anti-CTLA-4 antibody as well as anti-PD-1, have revolutionized the treatment of several cancers but fail to control cancer progression in a significant proportion of patients (102–104). In addition, primary resistance to ICI has been observed in other types of cancer, such as pancreatic cancer and glioblastoma (105–107). The current challenge consists of using combined therapy approaches to improve ICI efficacy (108). Recent findings indicate that CD4^+^ helper T cells can influence the response to immunotherapy in the success of ICI (109, 110). In patients with bone metastases of castration-resistant prostate cancer, increased Th17 instead of Th1 cells were found in bone metastases after ICI therapy and this correlated with a reduced efficacy of ICI compared to Th1 cells infiltration (111). Thus, these observations support the rational to combine ICI with epigenetic therapies that induce appropriate CD4^+^ helper T cells polarization in the TME (**Figure 3C**). In a murine hepatocellular carcinoma model, the administration of the HDACi belinostat improved the response to CTLA-4 inhibition. The production of IFN-γ by tumor-reactive CD8^+^ T cells was enhanced and the number of splenic Treg cells was decreased in mice treated with both CTLA-4 and belinostat combination (112). Moreover, the co-administration of the HDACi trichostatin A and anti-CTLA-4 could enhance the infiltration of CD4^+^ T cells and was associated with better antitumor effects (97) (**Figure 3C**). In a mouse model of lung cancer, the use of JQ1, a bromodomain-targeted BET inhibitor, could also enhance the response to anti-PD-1 antibodies by reprogramming CD4^+^ T cells in the TME. Indeed, JQ1 promoted an increase of Th1 cells at tumor site as well as depletion of Treg cells and improved survival compared to mice treated with ICI alone (113). Moreover, blocking EZH2 expression by the pharmacological inhibitor CPI-1205 in MB49 tumor-bearing mice was associated with a better survival when combined with anti-CTLA-4. These results were correlated with an increased infiltration of Th1 cells and cytotoxic effector T cells in the TME as well as a depletion of Treg cells (114). In mouse and human prostate cancer organoids models, inhibition of EZH2 increased the expression of the Th1 attracting-chemokines CXCL9 and CXCL10 and derepressed endogenous double-strand RNA (ds RNA) expression. Ds RNA expression thus triggered “viral mimicry pathway” and the expression of interferon-stimulated genes *via* the ds RNA sensor STING. EZH2 inhibition in murine prostate cancer cell lines with DZNep and EPZ also resulted in a drastic upregulation of Th1 cytokines TNF-α, IL-2 and IL-12. Overall, EZH2 inhibition increased CD4^+^ and CD8^+^ tumor infiltration in mice and potentiate prostate cancer response to anti-PD-1 therapy (115). Additionally, the HDACi CG-745 enhanced the anti-cancer effect of anti-PD-1 therapy in syngeneic tumor mouse models by remodeling the immune microenvironment. Indeed, CG-745 increased the proliferation of helper T cells, cytotoxic T cells and NK cells, while decreasing proliferation of regulatory T cells and inhibiting myeloid-derived suppressor cells as well as M2 macrophage polarization (116). In the triple-negative 4T1 breast cancer mouse model, HDACi enhanced the *in vivo* response to PD-1/CTLA-4 blockade. This effect was attributed to the up-regulation of PD-L1 and HLA-DR on tumor cells as well as the decrease of the recruitment of FOXP3^+^ CD4^+^ T cells in the TME (117). By modulating CD4^+^ T cells polarization and recruitment at tumor site, epigenetic therapies induce a favorable immune context in the TME that improve the response to ICI (**Figure 3C**). The role of HDACi as an immunomodulatory agent has been extensively studied (118, 119). However, there is limited knowledge concerning the effects of HDACi on T helper cells differentiation in clinical settings (120, 121). Example of epigenetic modulators currently approved or under clinical trials are listed in **Table 1**.

**Table 1:** Epigenetic modulators that may influence CD4^+^ T cells anticancer immunity.

Epigenetic Drug	Mechanism of action	Route of administration	Status	Cancer type	Adverse drug reaction (very common, all grades)
ZEN-3694	BETi	ORAL	Phase III	Prostate cancer	NCT02711956 (Phase I/II): decreased appetite, dysgeusia, fatigue, nausea, thrombocytopenia, visual symptoms
Guadecitabine	DNMTi	SC	Phase III	Acute Myeloid Leukemia	NCT02348489 (Phase I/II): anemia, febrile neutropenia, neutropenia, pneumonia thrombocytopenia, sepsis
Azacitidine	DNTMi	IV/SC	FDA/EMA approved	Myelodysplastic syndromes	arthralgia, anemia, anorexia, diarrhea, dizziness, epistaxis, febrile neutropenia, headache, hypokaliemia, infection, insomnia, leucopenia, neutropenia, pyrexia, thrombopenia, vomiting
				Chronic myelomonocytic leukemia	
				Acute myeloid leukemia	
Azacitidine (CC-486)	DNTMi	ORAL	FDA approved	Acute myeloid leukemia	abdominal pain, anorexia, arthralgia constipation, diarrhea, dizziness, fatigue nausea, febrile neutropenia, infections, vomiting
Decitabine	DNTMi	IV	FDA/EMA approved	Acute myeloid leukemia	anemia, diarrhea, epistaxis, febrile neutropenia, hepatic function abnormal, hyperglycemia, headache, infections, leucopenia, nausea, neutropenia, pyrexia, thrombocytopenia, vomiting
Decitabine and cedazuridine	DNTMi	ORAL	FDA approved	Myelodysplastic syndromes	arthralgia, constipation, diarrhea, dizziness, edema, fatigue, febrile neutropenia, headache, infections, hemorrhage, mucositis, myalgia, nausea, pyrexia
Tazemetostat	EZH2i	ORAL	FDA approved	Follicular lymphoma	anemia, anorexia, constipation, diarrhea, fatigue, headache, infections, lymphopenia, neutropenia, nausea, vomiting
Abexinostat	HDACi	ORAL	Phase III	Renal cell carcinoma	NCT01543763 (Phase I): anorexia, diarrhea, fatigue, hypertension, nausea, neutropenia, thrombocytopenia, vomiting
Belinostat	HDACi	IV	FDA approved	Peripheral T-cell lymphoma	anemia, constipation, diarrhea, dyspnea, edema, fatigue, headache, hypokalemia, increased blood lactate dehydrogenase, nausea, pyrexia, peripheral pruritus, prolonged QT, vomiting,
Entinostat	HDACi	ORAL	Phase III	Breast cancer	NCT01434303 (Phase I): anemia, diarrhea, fatigue, neutropenia, thrombocytopenia
Panabinostat	HDACi	ORAL	FDA/EMA approved	Multiple myeloma	anemia, diarrhea, dizziness, edema, fatigue, hypotension, hyponatremia, hypokaliemia, hypophosphatemia, headache, infections, leucopenia, neutropenia, nausea, thrombocytopenia, vomiting
Romidepsine	HDACi	IV	FDA approved	Cutaneous T-cell lymphoma	anemia, anorexia, hypomagnesemia, infections, leucopenia, nausea, neutropenia, pyrexia, thrombocytopenia, vomiting
				Peripheral T-cell lymphoma	
Tucidinostat	HDACi	ORAL	Approved outside the United-State and Europe	Breast cancer	NCT02482753 (Phase III): anorexia, anemia, diarrhea, hyperglycemia, hypokaliemia, hyperglycemia hypocalcemia, infections, leucopenia, nausea, neutropenia, thrombocytopenia, vomiting
				Peripheral T-cell lymphoma	
Vorinostat	HDACi	ORAL	FDA approved	Cutaneous T-cell lymphoma	anemia, anorexia, constipation, dizziness, diarrhea, fatigue, nausea, peripheral edema, thrombocytopenia, vomiting

Non-exhaustive list. BETi, Bromo and Extra Terminal domain inhibitor; DNMTi, DNA MethylTransferase inhibitor; EZH2i, Enhancer of zeste homolog 2 inhibitor; EMA, European Medicines Agency; FDA, Food and Drug Administration; HDACi, Histone deacetylase inhibitors; IV, Intravenous; SC, Subcutaneous.

## Discussion

CD4^+^ T cells mediate antitumor immune response and are involved in the response to anticancer immunotherapies. We have seen that epigenetics plays a key role in the regulation of CD4^+^ T cells differentiation, plasticity and memory formation. Moreover, we described how the TME could reprogram tumor reactive CD4^+^ T cells epigenetic landscape and modulate their functionality and recruitment at tumor site by regulating the expression of immune inflammatory cytokines and attracting chemokines. Epigenetics also control the expression of immune checkpoints, thus regulating tumor reactive CD4^+^ T cells exhaustion. Additionally, the conversion of Th17 cells to Treg cells in the TME was found to be regulated by miRNAs. Since the type of CD4^+^ T cell infiltrating the tumor can affect differently the prognosis of patients and the response to immunotherapy, epigenetic modulators can be used to induce appropriate CD4^+^ helper T cells polarization and recruitment in the TME. In this review, we presented recent literature showing the growing interest of combining epigenetic treatments with immunotherapy. Epigenetic therapies can decrease the number of Treg cells in the TME or their immunosuppressive capacities, therefore improving the response to immune checkpoint blockades. Th1 cells recruitment at tumor site can be enhanced by the epigenetically induced re-expression of endogenous retroviruses or Th1-attracting chemokines (CXCL9 and CXCL10) by tumor cells. The persistence and resistance to exhaustion of adoptively transferred CD4^+^ T cells in the TME can be preserved by epigenetic treatments. The combination of epigenetic therapies with anticancer vaccines can also promote the expression of proinflammatory genes by CD4^+^ T cells, thus enhancing the anticancer immune response. Therefore, the combination of epigenetic treatment and immunotherapy provides new insights in anticancer therapy. However, the role of epigenetics on the antitumor specific immune responses remains poorly characterized. This highlights the need to investigate the role of epigenetics in CD4^+^ T cells differentiation and plasticity in the TME. To bring answers to these issues, our team is currently screening hundreds of drugs targeting epigenetic enzymes to understand and modulate the polarization of expanded tumor infiltrating lymphocytes.

## Author Contributions

ER, MK, and OA wrote the manuscript. RL, EH, PP, and CB edited the manuscript. All authors contributed to the article and approved the submitted version.

## Funding

This work was supported by funding from institutional grants from INSERM, EFS and Univ. Bourgogne Franche-Comté and by the “Ligue Contre le Cancer”, the “Région Bourgogne Franche-Comté (projet d’envergure structurant C-ICI)”and european founds “Programme Interreg France-Suisse 2014-2020 (FEDER) – Projet R-TIC”.

## Conflict of Interest

The authors declare that the research was conducted in the absence of any commercial or financial relationships that could be construed as a potential conflict of interest.
